# Editorial: Women in sports 2022

**DOI:** 10.3389/fphys.2024.1367605

**Published:** 2024-02-13

**Authors:** Katia Collomp, Silvio Lorenzetti

**Affiliations:** ^1^ CIAMS, Université d’Orléans, Orléans, France; ^2^ CIAMS, Université Paris-Saclay, Orsay, France; ^3^ Sport, Physical Activity, Rehabilitation and Movement for Performance and Health Research Group, Orléans, France; ^4^ Laboratoire Anti-Dopage Français, LADF, Université Paris-Saclay, Orsay, France; ^5^ Swiss Federal Institute of Sport Magglingen, Magglingen, Switzerland

**Keywords:** female athlete, performance development, age, hormonal status, sport discipline, country, doping, diet

In the past, sports were primarily played by men. More recently, in addition to participating in sports for leisure and health purposes, more women are participating as professional athletes in high-performance sports. However, a gender gap in the prize money for many sports and a knowledge gap in sports science persist. Indeed, elite sports continue to distinguish between men and women, although sport is one of the most powerful platforms for promoting gender equality and empowering women and girls. To help women become elite athletes, researchers are working to answer some of the most important questions concerning not only the physiology of women in sports but also their behavior in relation to environmental factors.

The *Frontiers in Physiology* Research Topic on the research theme “*Women in Sports 2022*”includes a compilation of six articles on heterogeneous topics aimed at improving our knowledge of female athletes by exploring 1) the impact of age and training experience on performance development in competitive female athletes according to the sports discipline; 2) the links between sports discipline, training volume, contraceptive behavior, menstrual-cycle disorders or symptoms, and injuries; and 3) the use of different diets and prohibited substances among female athletes.

Performance development, participation, and relative age are important factors in women’s sports activities. Rüeger et al. present the longitudinal trends in young Swiss female long-jump athletes’ performance based on the valuable online database of Swiss Athletics, where data from over 16,000 athletes are included. In general, in the age categories U8–U17, the performance of athletes born between January and March was significantly better than that of athletes born between October and December. The authors suggest using the exact chronological age for a fair performance evaluation based on a clear age effect and the age-group limitation in their study to ensure long-term participation. The provided average performance-development data can be used for individual comparisons and personal development assessments.

van den Tillaar et al. evaluated the positions, sprint-skating performance, and playing-level of female professional bandy players. Bandy is the second most popular team winter sport after ice hockey and is played on a soccer-sized ice rink. A total of 74 bandy players were included and categorized according to their playing level and position. Overall, sprint-skating performance was influenced by the playing level, playing position, body mass, and training experience. Therefore, the authors suggest that junior-level female players, particularly offensive players, should spend more time training in sprint skating, and the development of acceleration abilities is crucial to reaching the elite level.

Baumgartner et al. address the important concept that female athletes exhibit different contraceptive behaviors, menstrual cycle disorders, and injuries across sports disciplines. In their survey of elite Swiss female athletes, half of the participants were aged <23 years, and 87% trained >10 h weekly. Primary amenorrhea typically occurs more frequently in female athletes with a low body mass index (Torstveit and Sundgot-Borgen, 2005; Oxfeldt et al., 2020). Compared with female athletes from other countries (Oxfeldt et al., 2020; Cheng et al., 2021), Swiss female athletes use fewer hormonal contraceptives (45.1%, mainly orally). In this study, athletes participating in lean sports were significantly less likely to use systemic hormonal contraceptives (25%) than those participating in non-lean sports. Moreover, athletes with a current or past episode of secondary amenorrhea and high training volumes (>20 h/week) exhibited a significantly higher risk of injury.

Bougault et al. examined the association between hormonal contraception and physical activity level and female students’ self-perception of menstrual symptoms. Oxfeldt et al. (2020) reported that combination hormonal contraceptive use lowers the perception of menstrual symptoms, regardless of the physical activity level, and intense physical activity (>10 h/week) significantly decreased the prevalence of both physical and mental symptoms in female students (Oxfeldt et al., 2020). Moreover, the impact of menses on physical and academic activities and on diet alterations was lesser in females with intense physical activity than in those who trained less. This effect was particularly evident in females not using hormonal contraceptives. However, the causal link remains unestablished.

Current recommendations for diet supplementation are largely based on studies in young, fit men, with a straightforward extrapolation to women. Hiroux et al. compared the effects of increased protein and ketone ester supplementation combined with a hypocaloric diet (70% of the optimal energy intake) on metabolism and performance in female athletes participating in recreational sports. The results showed that similar to their male counterparts, female athletes maintained their exercise capacity with the hypocaloric diet after both supplementations. In contrast to increased protein intake, ketone ester administration was unable to blunt muscle wasting. However, it improved overall stress parameters and preserved resting energy expenditure. Further studies are needed to determine the implicated mechanism(s) and possible extrapolation under extreme catabolic conditions often seen in elite female athletes.

Buisson et al. present a comparison between the anti-doping collections and prohibited substances detected in the Summer Olympics in Australia and New Zealand and those detected in France. In both geographical areas, the highest number of samples was collected from the same sports categories (mixed and endurance) and disciplines (athletics, aquatics, and cycling). Similarly, regardless of the region, more adverse analytical findings were observed in in-competition tests than in out-of-competition tests, with a larger number of detections in France before and after exemption for therapeutic use. The study highlighted a clear geographical and/or drug-availability impact with few substances in common. As with hormonal-contraceptive behavior, further studies are necessary to put these data into a global perspective, compare their use across countries, and explore possible new developments in the fight against doping among women.

In conclusion, sensitive and distinct tools with potential new markers need to be developed for female athletes owing to a comprehensive sex-specific exploration, considering the wide variations in sports disciplines, training load and experience, energy availability, and cultural factors linked to geographical regions. Although this Research Topic fails to close the gender gap, it highlights the importance and value of female-specific topics.
